# Leveraging Machine Learning to Combat Missingness and Error in Data

**DOI:** 10.23889/ijpds.v9i5.2518

**Published:** 2024-09-18

**Authors:** Beverley Phillips, Philip Witowski, Windra Sulaiman, Adam Ismail, Mark Sipthorp, Sharon Williams

**Affiliations:** 1 Centre for Victorian Data Linkage

## Objective

Poor quality data confounds efforts to link clients across datasets. To combat this, we have trialed an approach which seeks to identify linkage candidates using associations with related services.  We utilised a machine learning (ML) approach to query linkage candidates’ dataset associations and make predictions about whether candidates are likely to be a client of the service being integrated for linkage.

## Approach

Utilizing a K-nearest neighbors algorithm, we trained a model using 15 variables to predict whether a linkage candidate is a client of a family service dataset. We then evaluated the model's success, and modified its native performance to minimise false positive matches. Subsequently, we tested the validity of these predictions as linkage criteria by employing blocking strategies and linking the service dataset to our linkage spine. The trained model was then used to identify correct links against the spine.

## Results

The evaluation of the machine learning model yielded promising results, with high accuracy (88.5%) and precision (95.5%). Testing the predictions as linkage criteria resulted in highly accurate links ranging from 96.0% to 98.7% across different blocking strategies. Despite some records failing to establish any links to the spine, rates of false positive matches remained low (0.7% to 3.1%).

## Conclusion

Missingness and inaccuracy in data remains a key problem for data linkage, and a robust approach is required to resolve complex linkage cases. However, these findings suggest that machine learning can present novel options for a toolbox of many approaches to link problem records to a linkage spine.

